# Circular RNAs as promising biomarkers for human diseases: An update for 2018–2025

**DOI:** 10.1016/j.gendis.2026.102043

**Published:** 2026-01-19

**Authors:** Yiqing Chen, Shuting Guo, Dongchao Lu, Michail Spanos, Xuan Ye, Huihua Chen, Junjie Xiao

**Affiliations:** a School of Traditional Chinese Medicine, Shanghai University of Traditional Chinese Medicine, Shanghai 201203, China; b Institute of Cardiovascular Sciences, Shanghai Engineering Research Center of Organ Repair, Joint International Research Laboratory of Biomaterials and Biotechnology in Organ Repair (Ministry of Education), School of Life Science, Shanghai University, Shanghai 200444, China; c School of Integrative Medicine, Shanghai University of Traditional Chinese Medicine, Shanghai 201203, China; d Cardiovascular Division of the Massachusetts General Hospital and Harvard Medical School, Boston, MA 02114, USA; e Albert Einstein College of Medicine, Department of Internal Medicine, NCB, Bronx, NY 10461, USA

**Keywords:** Biomarker, circRNAs, Clinical translation, Diagnosis, Human diseases, Liquid biopsy, Prognosis

## Abstract

Circular RNAs (circRNAs) are a class of endogenous non-coding RNAs characterized by their unique covalently closed-loop structure. These molecules are key regulators of gene expression and are closely linked to various human diseases. Due to their intrinsic stability, evolutionary conservation, and tissue-specific expression, circRNAs have emerged as highly promising biomarkers for a wide range of diseases. This review comprehensively synthesizes progress in research from 2018 to 2025 on the biogenesis, nuclear export, and functional mechanisms of circRNAs in human diseases. Further, we update novel detection technologies and methods of circRNAs and discuss the prospects and challenges of translating circRNAs into clinical biomarkers, aiming to facilitate clinical applications.

## Background

Circular RNAs (circRNAs) are a class of endogenous non-coding RNAs defined by their covalently closed continuous loop structure. This distinctive topology, lacking the 5′ cap and 3′ poly(A) tail characteristic of linear RNAs, sets them apart from their conventional counterparts.^1^ Initially identified in 1976 in plant-pathogenic viroids, circRNAs were once thought to be rare byproducts of aberrant RNA splicing.^2^ The increasing identification of circRNAs in recent years is largely driven by two technical advances. High-throughput sequencing technologies enable large-scale RNA profiling and computational methods specifically optimized for back-splice junction detection that distinguish circRNAs from linear RNAs. Thus, circRNAs have been increasingly identified to exhibit specific abundance differences in various human diseases and play key regulatory roles in multiple pathophysiological processes.^3^ Furthermore, the covalent closed-loop structure of circRNA confers exceptional stability, enabling effective resistance to ribonuclease degradation, while evolutionary conservation ensures consistent expression patterns across different individuals. These properties collectively establish circular RNA as a robust and reliable molecular biomarker for bodily fluid detection.^4^

In 2018, our group published a review that summarized the roles of circRNAs in disease progression and discussed their potential as biomarkers for various human diseases.^5^ The subsequent years have witnessed a substantial surge in research on circRNAs, driven by advanced bioinformatics and emerging novel technologies. This review, accordingly, serves not merely as a chronological follow-up but as a comprehensive synthesis that summarizes the research progress achieved since 2018. We specifically focus on updated insights into circRNA biogenesis and disease-related functions, the latest advancements in detection methodologies, as well as the prospects and challenges of clinical translation of circRNA as biomarkers, offering a valuable reference for the field. Full methodological details, including the search strategy and screening process, are provided in Figure S1.

## Biogenesis, nuclear export, and functions of circRNAs

### Biogenesis of circRNAs

CircRNAs are generated by a distinctive back-splicing mechanism.^6^ This process requires the spatial proximity of a downstream 5′ splice donor site to an upstream 3′ splice acceptor site, facilitated either by base pairing between complementary intronic sequences or by dimeric RNA-binding proteins (RBPs) such as QKI and MBL, which bridge the intervening intronic elements.^7^, ^8^, ^9^ The resulting reverse splicing reaction covalently joins these sites, forming a single-stranded, closed circular molecule from pre-mRNA. The splicing mechanism removes introns using the spliceosome, ligating the remaining exon sequences that may contain protein-coding information. This process generates three primary subtypes of circRNAs through specific back-splicing mechanisms: exonic circRNAs, formed by back-splicing that connects a 5′ splice donor to a 3′ splice acceptor; intronic circRNAs, derived from intron lariats that escape debranching during linear splicing; and exon-intron circRNAs (EIciRNAs), which retain intronic sequences between circularized exons and enhance cis-regulation of parental gene expression.^10^, ^11^, ^12^, ^13^

Under pathological conditions, alternative splicing pathways can generate diverse classes of circRNAs. One notable subtype is read-through circRNAs, which are produced due to failed transcription termination followed by reverse splicing.^14^ Another pathological subtype, fusion circRNAs, associated with chromosomal translocations in cancer, show resistance to apoptosis-inducing drug treatments and enhance cellular transformation and survival.^15^ In addition, tRNA intronic circRNAs are a distinct class of circRNAs spliced from the intronic regions of precursor tRNAs during their maturation^16^ (Fig. 1). Future advancements in biotechnological and bioinformatics strategies will enhance our understanding of circRNA complexity.

**Figure 1:**
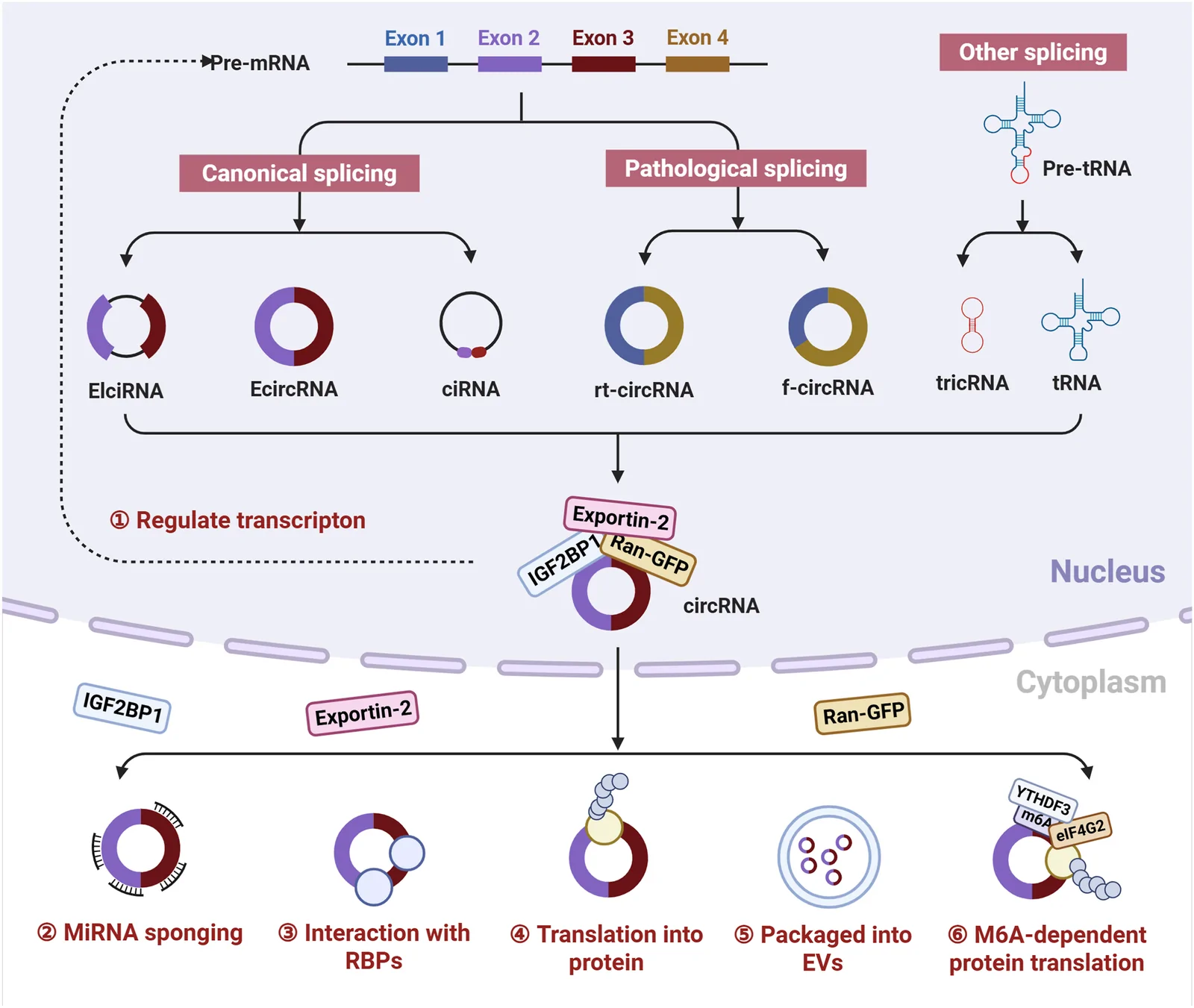
Overview of circRNA biogenesis, nuclear export, and functions. The schematic illustrates the multiple pathways of circRNA biogenesis, including canonical splicing, pathological splicing, and other splicing. circRNA exerts its regulatory functions by transcriptional regulation, miRNA sponging, RBP interaction, protein translation, exosomal packaging, and m^6^A modification and recognition. The figure was created with BioRender.com, with permission.

### Nuclear export of circRNAs

Unlike linear mRNAs, the nuclear export pathway for circRNAs remains poorly understood. Recent studies have emphasized the importance of the small GTPase Ran (Ran-GTP) in facilitating the directional nuclear export of circRNAs. The adaptor protein insulin-like growth factor 2 mRNA binding protein 1 (IGF2BP1) mediates the interaction with nuclear export protein-2 (Exportin-2) and promotes the assembly of a nuclear export complex containing the transporter chromosomal region maintenance 1 (CRM1). This complex mediates circRNA movement into the cytoplasm, allowing their functional engagement in the cytoplasmic environment.^17^

### Functions of circRNAs

As shown in Figure 1, the functions of circRNAs can be broadly categorized into several groups.

**Regulation of parental gene transcription:** EIciRNAs are characterized by the presence of intronic sequences derived from their parental genes, and they play a pivotal role in enhancing gene transcription. This enhancement is facilitated through the binding of EIciRNAs to U1 snRNP via RNA–RNA base pairing, resulting in the formation of an EIciRNA-U1 snRNP complex. This complex subsequently interacts with RNA polymerase II at gene promoters to facilitate transcription.^18^ Analogously, intronic circRNAs that accumulate at their sites of transcription serve as molecular tethers that stimulate Pol II elongation, leading to enhanced expression of their parental genes.^19^^,^^20^

**MicroRNA (miRNA) sponges:** miRNAs are pivotal post-transcriptional regulators with broad impacts on gene expression in both physiological and pathological contexts. By fine-tuning gene networks, miRNAs directly connect molecular mechanisms to clinical translation, rendering them essential for elucidating disease progression and advancing precision medicine.^21^ circRNAs function as sponges for miRNAs, competing for their binding sites and counteracting their inhibitory effects on mRNAs.^22^ This mechanism is particularly important in oncology, as it often leads to the derepression of oncogenes or tumor suppressors (Fig. 2). Although miRNA sponging is a well-established function of circRNAs, this mechanism is not universal and likely applies to a specific subset. Many circRNAs operate through alternative mechanisms to exert their biological effects.

**Figure 2:**
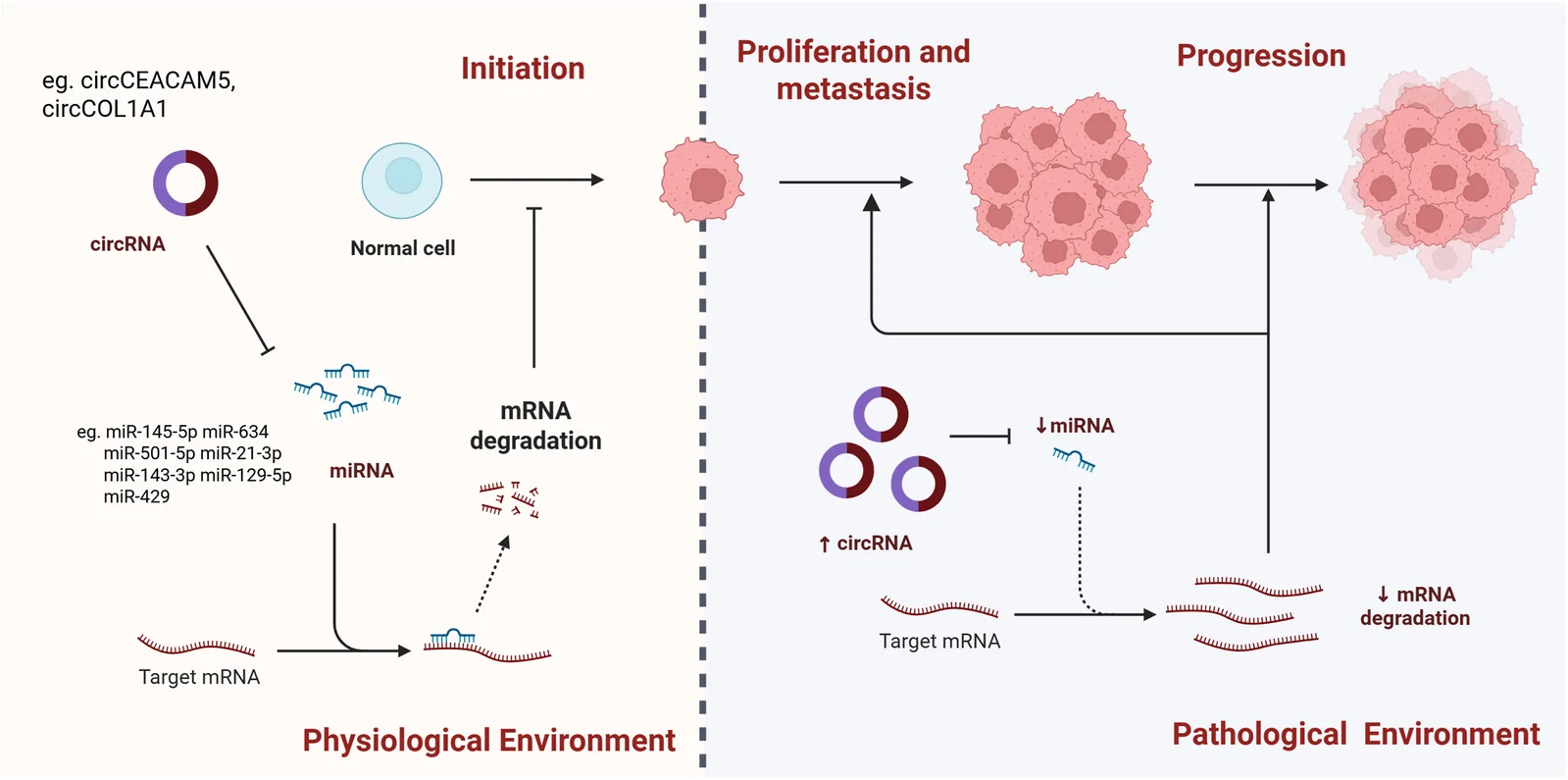
Mechanisms of circular RNAs functioning in physiological and pathological environments. The schematic illustrates the mechanisms by which circRNAs function in physiological and pathological environments, taking cancer as an example. In the pathological environment of cancer, altered circRNA (*e.g.*, circCEACAM5, circCOL1A1) levels influence processes like cell proliferation and metastasis by modulating miRNA (*e.g.*, miR-145-5p, miR-634)-mediated target mRNA regulation. The figure was created with BioRender.com, with permission.

**Protein translation:** Certain circRNAs can be translated into proteins despite lacking a free 5′ end, which is indispensable for canonical eukaryotic initiation. This translation can occur via mechanisms like rolling circle amplification, thereby dispensing with the requirement for IRES, poly(A) tails, or 5′ cap structures.^23^^,^^24^

**RBPs as sequestering agents:** Some circRNAs bind to RBPs, inhibiting the transport of these proteins to the nucleus and preventing their cellular localization. For instance, circ-FOXO3 binds to the inhibitor of DNA binding 1 (ID1), early 2 factor 1‌ (E2F1), and hypoxia inducible factor 1 α (HIF1-α), preventing their nuclear localization and thereby redirecting their cellular localization, which affects various cellular functions.^25^

**Packaged into extracellular vehicles (EVs):** EVs are bilayer vesicles measuring 40–100 nm in size, released by cells into the extracellular matrix. They serve as important messengers that mediate intercellular communication and have also been utilized in the development of biomarkers. circRNAs can be encapsulated into these vesicles, allowing them to mediate both proximate and remote intercellular communications and play a key role in regulating key aspects of disease progression, including proliferation, angiogenesis, metabolism, and immune escape.^26^

**m^6^A-dependent protein translation:** Upon recognition by the m^6^A reader YTH N6-methyladenosine RNA binding protein 3 (YTHDF3), m^6^A-modified circRNAs initiate translation via recruitment of eukaryotic translation initiation factor 4G2 (eIF4G2). This pathway establishes a dynamic regulatory axis, being positively regulated by the methyltransferase like 3 (METTL3) and methyltransferase like 14 (METTL14) and negatively by the demethylase, fat mass and obesity-associated protein (FTO).^27^ Recent research has highlighted novel functions of circRNAs, including acting as protein decoys, scaffolding for protein recruitment, and modulators of protein function through isolation or proximity enhancement. However, these roles are still under investigation and require further verification.^28^

### Potential of circRNAs as biomarkers of human diseases

Biomarkers are biochemical indicators that objectively reflect physiological or pathological processes, serving crucial roles in early disease diagnosis, staging, prognosis monitoring, and treatment evaluation. Human diseases, especially cancer, due to the lack of obvious early symptoms and traditional circulating or tissue biomarkers, often suffer from low sensitivity and specificity, highlighting an urgent need for novel biomarkers.^29^, ^30^, ^31^ Advances in sequencing technologies and detection methods have positioned circRNAs as promising biomarker candidates, attracting growing research interest.^32^ Among non-coding RNAs, circRNAs are notable for their structural robustness due to the absence of 5′ caps and 3′ poly(A) tails, rendering them inherently resistant to RNase R. This intrinsic stability, combined with their dynamic and tissue-specific expression, establishes them as highly promising biomarkers for a wide range of human diseases.^5^^,^^33^, ^34^, ^35^ Notably, pre-analytical variability in circulating circRNA studies, such as tube type selection, centrifugation timing, freeze–thaw cycles, and extraction methods, significantly impacts detection accuracy. Standardization remains challenging due to the absence of stable reference genes and the variable specificity of quantitative PCR, RNA sequencing, and droplet digital PCR platforms in distinguishing circular from linear RNAs. Addressing these issues through unified protocols is crucial for realizing the clinical potential of circulating circRNAs. Table 1 provides a summarized representation of updated circRNAs with the potential to become biomarkers for human diseases between 2018 and 2025.

**Table 1:** Summary of dysregulated circRNAs in various human diseases.

Disease	circRNA	Expression	Study type	Sample type	Sample sizes	AUC	Reference
Gastric cancer	circCEACAM5	Up	Cross-sectional study	Tissue	5 patients with gastric cancer	0.96	^36^
	circCOL1A1					0.94	^37^
	circ_0007507	Up	Cross-sectional study	Tissue and serum	100 patients with gastric cancer and 80 healthy controls	0.832	
	circ_0045602, circ_0008768, circ_0001013, circ_0007380, circ_0034398, circ_0052001, circ_0002019, circ_0006089	Up	Cross-sectional study	Serum	194 patients with gastric cancer and 94 healthy controls	Combined: 0.87 in training set; 0.83 in validation set	^38^
Pancreatic cancer	circ_0060733, circ_0006117, circ_0064288, circ_0007895, circ_0007367	Down	Cohort	Tissue and serum	158 patients with pancreatic cancer and 81 healthy controls	Combined: 0.83 in training cohort; 0.82 in validation cohort	^39^
	circ_0006220	Up	Cross-sectional study	Extracellular vehicles from serum	62 patients with pancreatic cancer and 62 healthy controls	0.7817	^40^
	circ_0001666					0.8062	
	circ_001569	Up	Cross-sectional study	Serum	26 patients with pancreatic cancer and 97 healthy controls	0.716	^41^
Hepatocellular carcinoma	circ_0005397	Up	Cohort	Extracellular vehicles from serum	89 patients with hepatocellular carcinoma, 40 patients with benign liver disease, and 79 healthy controls	0.737	^44^
	circ-ZEB1	Up	Cross-sectional study	Tissue	56 patients with hepatocellular carcinoma	/	^106^
	circ_0004001, circ_0004123, circ_0075792	Down	Cross-sectional study	Serum	71 patients with hepatocellular carcinoma and 40 healthy controls	Combined: 0.89	^45^
Colorectal cancer	circ_0082182	Down	Cross-sectional study	Serum	156 patients with colorectal cancer and 66 healthy controls	Combined: 0.8346	^47^
	circ_0000370	Down	Cross-sectional study	Serum	156 patients with colorectal cancer and 66 healthy controls	Combined: 0.8346	^47^
	circ_0035445	Up					
	circGPRC5A	Up	Cross-sectional study	Tissue	80 patients with colorectal cancer	/	^48^
Non-small cell lung cancer	circ_0023179	Up	Cross-sectional study	Serum	126 patients with non-small cell lung cancer and 114 healthy controls	0.831	^50^
	circFOXP1	Up	Cross-sectional study	Serum	82 patients with non-small cell lung cancer and 24 healthy controls	0.88	^51^
Clear cell renal carcinoma	circCHST15	Up	Cross-sectional study	Tissue	175 patients with clear cell renal carcinoma	/	^52^
Cervical cancer	circ_0000285	Up	Cross-sectional study	Serum and cervical exfoliated cells	65 patients with early-stage cervical cancer, 87 patients with locally advanced cervical cancer, and 116 healthy controls	0.855	^53^
Breast cancer	circCAPG	Up	Cross-sectional study	Tissue	132 breast cancer patients	/	^55^
Ovarian cancer	circ_0003972	Down	Cross-sectional study	Tissue and serum	41 ovarian cancer patients	0.724	^56^
	circ_0007288	Down				0.790	
Bladder cancer	circ_102336	Up	Cross-sectional study	Tissue	64 bladder cancer patients	/	^57^
Coronary artery disease	circ_0001879	Up	Cross-sectional study	Serum	24 patients with coronary artery disease and 7 healthy controls	0.703	^67^
	circ_0004104					0.700	
Hypertension	circ_0037911	Up	Cross-sectional study	Serum	100 patients with essential hypertension and 100 healthy controls	0.627	^68^
	circ_0126991	Up	Cross-sectional study	Serum	89 patients with essential hypertension and 89 healthy controls	0.774	^69^
	circ_0014243	Up	Cross-sectional study	Serum	89 patients with essential hypertension and 89 healthy controls	0.732	^70^
Alzheimer's disease	circRNA_050263	Up	Cross-sectional study	Serum	21 Alzheimer's disease patients, 5 mild cognitive impairment patients, and 24 healthy controls	0.980	^75^
	circRNA_403959	Up				0.714	
	circRNA_003022	Up				0.710	
Parkinson's disease	circEPS15	Down	Cross-sectional study	Serum	89 Parkinson's disease patients	0.9612	^76^
Diabetes mellitus	circRNA_100632	Up	Cross-sectional study	Peripheral blood mononuclear cells	75 diabetes patients and 115 healthy controls	0.846	^77^
	circRNA_405498, circRNA_100033	Down	Cross-sectional study	Peripheral blood mononuclear cells	81 type 1 diabetes patients and 143 healthy controls	Combined: 0.992 in training set; 0.995 in validation set	^78^
	circRNA_0039480, circRNA_0026497	Up	Cohort	Extracellular vehicles from plasma	81 patients with gestational diabetes mellitus and 143 healthy controls	Combined: first trimester 0.875, second trimester 0.929, third trimester 0.714	^79^
Hepatitis B virus (HBV)	circPanel	Up	Cross-sectional study	Tissue and serum	52 patients with chronic HBV, 50 patients with liver cirrhosis, 158 patients with HBV-HCC, and 53 healthy controls	0.863 in training set; 0.843 in validation set 1 and 0.864 in validation set 2	^80^
Tuberculosis	circ_0028883	Down	Cross-sectional study	Peripheral blood mononuclear cells	20 patients with active tuberculosis and 20 healthy controls	0.773	^81^
Ankylosing spondylitis	circ_102532	Up	Cross-sectional study	Serum	60 patients with ankylosing spondylitis and 30 healthy controls	0.642	^82^
	circ_001544	Up	Cross-sectional study	Serum	60 patients with ankylosing spondylitis and 30 healthy controls	0.720	^82^
	circ_012732	Up				0.550	
Myasthenia gravis	circ_5333-4	Down	Cross-sectional study	Serum	3 patients with myasthenia gravis and 3 healthy controls	0.864	^83^

### circRNAs as biomarkers for cancer

Cancer is more often detected at advanced stages, typically when metastatic dissemination has occurred. This process entails a multi-step cascade, beginning with the detachment of malignant cells from the primary tumor, followed by local invasion, intravasation into the circulation, evasion of immune surveillance, and culminating in the colonization and outgrowth at distant sites. circRNAs, due to their structurally stable nature, exhibit specific expression profiles throughout various stages of tumor progression. These RNAs can be consistently detected in tissues as well as in plasma, serum, and other bodily fluids. Owing to these properties, circRNAs are promising candidates for effective biomarkers that could greatly improve early cancer detection and prognostic assessments.

**Gastric cancer (GC)**: GC is the third leading cause of cancer mortality globally and is known for its aggressive nature. Although diagnostic and therapeutic strategies have advanced, treatment options for advanced-stage GC patients are still limited, leading to poor prognoses and negligible improvements in survival. The discovery of effective biomarkers is therefore essential to advancing GC management. The area under the receiver operating characteristic (ROC) curve (AUC) for circCEACAM5 and circCOL1A1 in GC patients was notably high at 0.96 and 0.94, respectively. These results underscore their potential clinical utility as valuable diagnostic biomarkers.^36^ Integrating circRNAs with traditional biomarkers significantly improves the sensitivity, specificity, and accuracy of GC diagnosis and prognosis. A notable example is serum circ_0007507, which demonstrates differential expression across treatment-naïve GC patients, post-operative individuals, and cases of recurrence, underscoring its promise as a versatile biomarker for diagnosis and disease monitoring. The AUC for serum circ_0007507 in GC patients was determined to be 0.832, which increased to 0.849 when combined with conventional tumor markers, such as carcinoembryonic antigen (CEA) and carbohydrate antigen 199 (CA199), thereby enhancing its diagnostic value.^37^ In addition, a combination of 8-circRNA biomarkers based on tissue and serum samples from GC patients can robustly and specifically identify early-stage GC, serving as an effective non-invasive biomarker for liquid biopsies.^38^

**Pancreatic cancer (PDAC)**: PDAC is characterized by a very low survival rate due to delayed symptom presentation and a lack of specific diagnostic markers, often being too late for detection in clinical practice. circRNAs such as circ_0060733, circ_0064288, circ_0006117, circ_0007367, and circ_0007895 are stably expressed in early-stage PDAC, exhibiting higher specificity than conventional biomarkers for identifying patients at an early stage.^39^ Furthermore, circ_0006220 and circ_0001666 are abundant in serum EVs, and circ_001569 is elevated in PDAC patient serum, collectively highlighting their potential as novel diagnostic biomarkers.^40^^,^^41^

**Liver cancer:** Liver tumors are a leading cause of global cancer mortality. While management has improved, current biomarkers lack sufficient sensitivity and specificity, creating a critical need for more reliable tools for early detection and surveillance.^42^ Growing evidence underscores the significant roles of circRNAs in liver tumorigenesis and metastasis, establishing their strong potential as biomarkers for diagnosing and prognosticating liver tumors.^43^ The sensitivity of plasma circ_0001445 in detecting hepatocellular carcinoma (AUC = 0.89) was significantly better than that of the traditional biomarker alpha-fetoprotein (AFP), and it is expected to be a biomarker for early serologic detection of hepatocellular carcinoma occurrence and progression, while serum exosome-derived circ_0005397 was reported to be significantly improved diagnostic sensitivity and specificity in combination with traditional markers.^44^ CircZEB1 was strongly correlated with advanced metastasis and poor prognosis in hepatocellular carcinoma. In contrast, circ_0004001, circ_0004123, and circ_0075792 were significantly down-regulated in patient serum (AUC = 0.89).^45^ These findings collectively highlight the promise of these circRNAs as novel biomarkers for early cancer detection and treatment monitoring.

**Colorectal cancer (CRC):** Accumulating evidence indicates that circRNAs are dysregulated in CRC tissues, cells, EVs, and blood.^46^ circ_0082182 and circ_0000370 are up-regulated in CRC patient plasma, contrary to the down-regulation of circ_0035445,^47^ while elevated tissue levels of circGPRC5A are associated with larger tumor size, advanced stage, and lymph node metastasis.^48^ Collectively, these biomarkers represent promising tools for the early diagnosis and prognostic assessment of colorectal cancer.

**Lung cancer:** Lung cancer continues to exhibit high morbidity and mortality rates worldwide, significantly burdening affected patients. Non-small cell lung cancer comprises approximately 85% of all lung cancer cases and is uniformly associated with poor survival outcomes.^49^ circRNAs act as central regulators that drive critical malignant phenotypes in non-small cell lung cancer, such as cell proliferation, migration, and invasion. circ_0023179 and circFOXP1 were found to be highly expressed in patient serum, with higher sensitivity and specificity compared with traditional tumor marker CEA, cytokeratin 19 fragment antigen 21-1 (CYFRA21-1), and squamous cell carcinoma antigen (SCC), establishing them as ideal biomarkers for prognostic monitoring in non-small cell lung cancer.^50^^,^^51^

**Clear cell renal carcinoma:** Metastasis is the primary cause of mortality in patients with clear cell renal carcinoma. Thus, the inherent stability and tissue-specific expression of circRNAs make them favorable prognostic biomarkers for the disease. Elevated circCHST15 expression in clear cell renal carcinoma tissues correlates with aggressive clinicopathological features and serves as a robust independent predictor of reduced overall and disease-free survival following surgical resection.^52^

**Cervical cancer**: Cervical cancer is an important causative factor of cancer-related deaths in women worldwide. The search for effective early biomarkers is of great significance for precision diagnosis and treatment. Preoperative serum and cervical exfoliated cell expression of circ_0000285 was elevated in cervical cancer patients, and postoperative serum circ_0000285 expression levels were significantly correlated with tumor recurrence.^53^ The up-regulation of circ_0018289 expression was significantly correlated with malignant phenotypes such as depth of tumor invasion and vascular infiltration, and its dynamic expression profile may be a potential molecular target for predicting lymph node metastasis and therapeutic response by regulating oncogenic signaling pathways such as Wnt/β-catenin.^54^ These findings reveal the critical role of circRNAs in the development of cervical cancer and provide a new perspective for the construction of a multidimensional biomarker monitoring system.

**Other cancers:** The clinical management of breast cancer is challenged by its heterogeneity and the scarcity of defined molecular targets. In this context, a decade-long follow-up study revealed that circCAPG was significantly up-regulated in triple-negative breast cancer tissues compared with normal counterparts, highlighting its potential relevance.^55^ Additionally, its expression was associated with improved overall survival, supporting its potential as a favorable prognostic biomarker.^55^ Furthermore, studies on various cancers, including ovarian cancer^56^ and bladder cancer,^57^ have reported the presence of differentially abundant circRNAs in cancerous tissues or body fluids. These circRNAs demonstrate considerable promise as biomarkers for cancer diagnosis, prognosis, and treatment guidance, underscoring their broad and growing significance across diverse cancer types. However, most current findings rely on single-center retrospective data that lack prospective validation. Furthermore, studies often fail to adequately adjust for confounding factors, and substantial sample heterogeneity undermines cross-study consistency. Moreover, technical challenges in blood fractionation^58^ and EV isolation, such as platelet contamination, introduce substantial bias. This is particularly relevant given that EVs, while capable of stabilizing circRNAs, require standardized and reproducible isolation methods to become reliable tools in research and diagnostics.

### circRNAs as biomarkers for cardiovascular diseases

Cardiovascular diseases represent the leading cause of global morbidity and mortality, encompassing conditions such as myocardial ischemia-reperfusion injury, myocardial infarction, atherosclerosis, and heart failure.^59^^,^^60^ Traditional biomarkers, including cardiac troponin, have been instrumental in detecting cardiovascular injuries; however, they face challenges in specificity and sensitivity.^61^^,^^62^ Given the practical difficulties in obtaining cardiac tissue biopsies, circulating biomarkers like circRNAs present a reliable alternative for early diagnosis and monitoring of cardiovascular diseases due to their stability and tissue-specific abundance changes.^32^ An increasing number of studies have highlighted circRNAs' critical role in regulating various pathological processes of cardiovascular diseases, including cardiomyocyte hypertrophy, fibrosis, autophagy, and apoptosis.^63^, ^64^, ^65^, ^66^ Detecting circRNA expression in the peripheral blood of coronary artery disease patients has identified circ_0001879 and circ_0004104 as novel diagnostic biomarkers.^67^ Bioinformatic analysis of plasma circRNA expression profiles in hypertensive patients revealed circ_0037911,^68^ circ_0126991,^69^ and circ_0014243^70^ as promising biomarkers for this hypertension. However, the specificity of circRNA biomarkers is often compromised by systemic conditions. For instance, post-myocardial infarction inflammation or comorbidities can alter circRNA expression in non-cardiac tissues. circ_0001900 is up-regulated in atherosclerosis but has also been observed in CRC.^71^^,^^72^

Additionally, with advances in cancer management, the intersection of cancer and cardiotoxicity mediated by anti-cancer therapies, such as cyclophosphamide or anthracyclines, has become increasingly relevant. These treatments are known for their serious cardiotoxic side effects, which can limit therapeutic dosing and overall cancer management. circRNAs, significant in both cancer and cardiovascular biology through their roles in proliferation, metastasis, and drug resistance, are anticipated to serve as monitoring biomarkers for cardiac side effects of cancer therapy. Nonetheless, this potential requires further validation through extensive clinical cohorts and foundational studies.^73^^,^^74^

### circRNAs as biomarkers for neurodegenerative diseases

Neurodegenerative diseases, characterized by neuronal damage and death, are difficult to diagnose early and lack effective treatments. Due to the insidious symptoms in the early stage of the disease, traditional biomarkers such as tau protein and amyloid in cerebrospinal fluid have some diagnostic value; however, their invasive detection methods and lack of sensitivity limit their clinical application. circRNAs can be stabilized in the cerebrospinal fluid via EVs, providing the possibility of noninvasive detection.

The potential of circRNAs as early diagnostic indicators is exemplified in Alzheimer's disease, where circRNA_050263, circRNA_403959, and circRNA_003022 are significantly up-regulated in patient cerebrospinal fluid, especially at the mild cognitive impairment stage. This pre-dementia expression highlights their promise for tracking initial neurodegenerative progression.^75^ In terms of specificity, circEPS15 exhibits a distinct expression profile that aids in differentiating Parkinson's disease from other movement disorders such as multiple system atrophy and essential tremor. Plasma levels of circEPS15 are specifically decreased in Parkinson's disease patients and correlate with motor symptom severity, highlighting its utility in improving diagnostic accuracy and serving as a companion biomarker for Parkinson's disease.^76^ In the future, individualized combinations of circRNA markers can be developed by combining patients' genetic backgrounds and epigenetic features for precise typing and therapeutic guidance.

### circRNAs as biomarkers for metabolic diseases

CircRNAs show promise as biomarkers for metabolic diseases. In type 1 diabetes mellitus, circRNA_100632, circRNA_405498, and circRNA_100033, studied retrospectively in peripheral blood mononuclear cells, had high diagnostic AUCs.^77^^,^^78^ A plasma EV circRNA panel from a gestational diabetes mellitus cohort revealed that circRNA_0039480 and circRNA_0026497 were robustly up-regulated across disease stages, supporting their utility as candidate biomarkers for the early, non-invasive diagnosis of gestational diabetes mellitus.^79^ However, most existing studies are limited by their retrospective nature, inadequate sample sizes, and a lack of prospective validation, which constrains their clinical applicability. Therefore, future multi-center, prospective studies are required to verify these findings.

### circRNAs as biomarkers for other diseases

In infectious diseases, circRNAs also show biomarker potential. For hepatitis B virus infection, circPanel is aberrantly expressed in the serum of hepatitis B virus-infected patients and correlates with viral load.^80^ In tuberculosis, circ_0028883 in peripheral blood mononuclear cells exhibits distinct expression patterns between active tuberculosis patients and healthy controls, with promising diagnostic AUC values, hinting at utility for early detection.^81^ circ_102532, circ_001544, and circ_012732 are significantly overexpressed in peripheral blood mononuclear cells of ankylosing spondylitis patients. Their expression levels strongly correlate with disease activity, consequently highlighting their potential as biomarkers for diagnosis and monitoring.^82^ Furthermore, circ_5333-4 is dysregulated in the peripheral blood of myasthenia gravis patients, suggesting its utility as a diagnostic and monitoring tool for this disease.^83^ However, like in other disease areas, related circRNA biomarker studies are retrospective and lack large-scale prospective validation.

### Advances in circRNA detection methods

With the advancement of detection technology, non-coding RNAs, once considered as “transcriptional noise”, have received more and more attention from researchers and scholars, and have become hotspots for medical research and promising candidates for effective biomarkers. Among non-coding RNAs, circRNAs possess a unique covalently closed loop structure that lacks 5′ caps and 3′ poly(A) tails. This structure confers exceptional stability and high conservation, thereby rendering them inherently resistant to RNase R degradation. This, coupled with their tissue-specific abundance changes during development in multiple tissues, gives circRNAs great potential as biomarkers in diagnosing human diseases and forecasting clinical outcomes. Conventional circRNA detection methods, such as Northern blotting, can be used to assess the presence and size of circRNAs with low sensitivity, whereas strand shifting and rolling-cycle replication of quantitative PCR during reverse transcription can lead to the generation of multiple repetitive cDNA copies for a single circRNA, resulting in inaccurate results in the determination of circRNA concentration. These limit the use of circRNAs in routine clinical diagnostics. Based on this, we updated the summary of technological advances that can be used for circRNA detection, with a view to providing a reference for the development of more efficient and cost-effective circRNA detection methods (Fig. 3).

**Figure 3:**
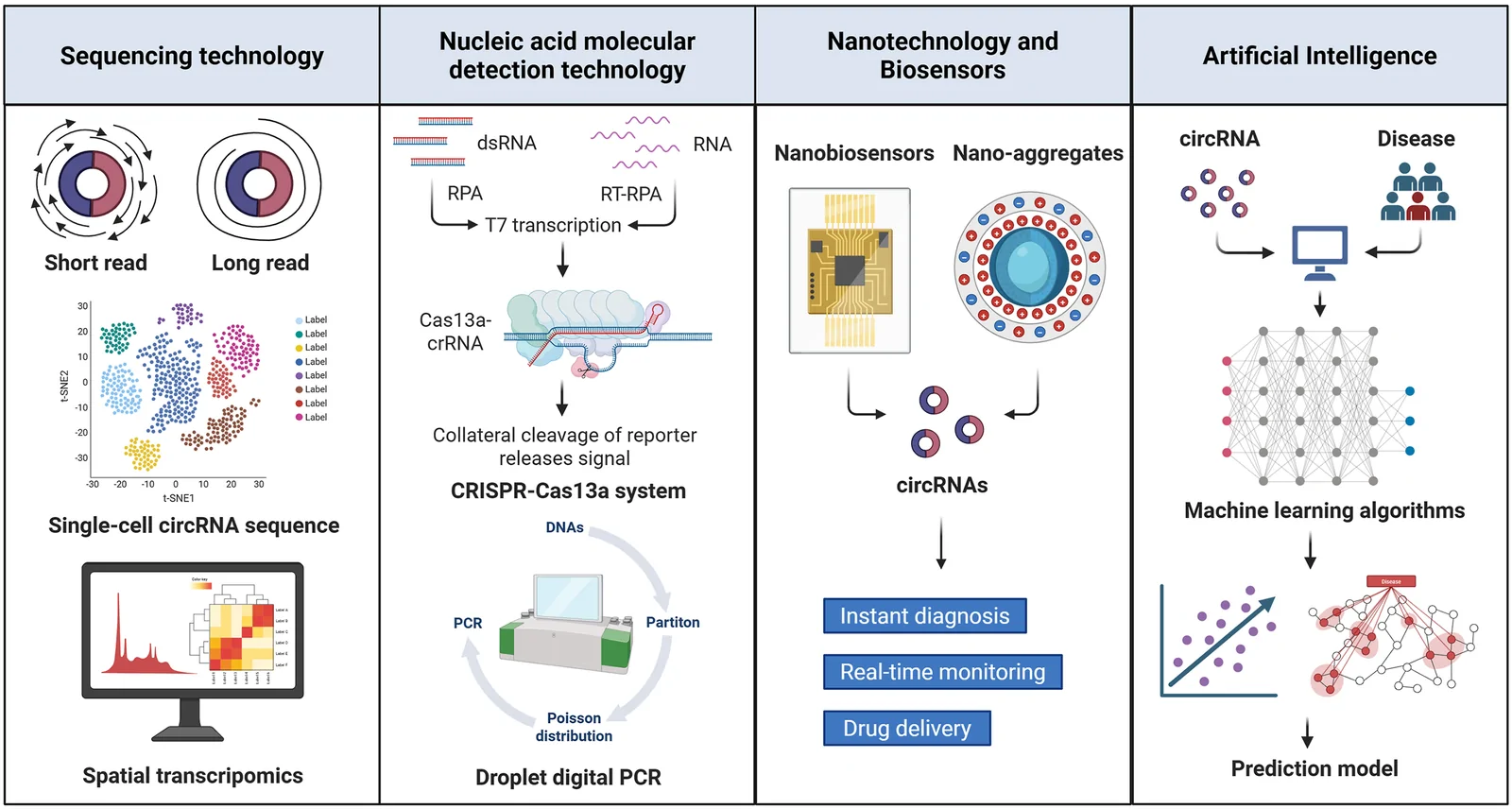
Overview of advances in circRNA detection methods. The schematic illustrates the innovations in sequencing technology, nucleic acid molecular detection technology, nanotechnology and biosensors, and an AI-based disease prediction model. The figure was created with BioRender.com, with permission.

### Innovations in sequencing technology

**Short-read short and long-read long sequencing:** As shown in Table 2, efficient detection of circRNA depends on the development of high-throughput sequencing technologies. RNA-sequencing data analysis needs to rely on specific algorithms, such as CIRCexplorer and CIRI2,^84^ to identify back-splice junction (BSJ). However, a major challenge confronting these tools is the prevalence of false positives in BSJ detection. Short sequencing reads can align non-specifically to repetitive genomic regions or generate artifactual BSJ signals due to RNA degradation. The next-generation sequencing technology, short-read-length sequencing, which is most notably characterized by high throughput and short read lengths, directly identifies circRNA-specific splicing through paired-end reads spanning the reverse splice site. Alternatively, circRNAs can be detected by indirectly comparing exon coverage between samples treated with RNase R to degrade linear RNAs and untreated controls. Conventional short read lengths, although capable of obtaining a large amount of data in a single experiment, may pose splicing difficulties and accuracy challenges when reconstructing genomes with long sequences or the presence of repetitive regions. The development of third-generation sequencing technology, long-read sequencing, has solved these problems well, mainly utilizing the strand replacement activity of reverse transcriptase to perform roll-over reverse transcription to generate DNA copies of multiple associates of a single circRNA. The circRNA-derived cDNA is then ligated into a complete loop structure and amplified by rolled-loop amplification, which greatly improves the accuracy of circRNA detection. However, long-read RNA sequencing can only process a small number of samples per run, rendering it unsuitable for large-scale cohort screening. Furthermore, the substantially higher cost of long-read sequencing per sample compared with short-read RNA sequencing poses a major barrier to its broad clinical adoption.^85^^,^^86^

**Table 2:** Summary of technologies used for the quantification and analysis of circRNAs.

Detection method	Mechanism	Advantage	Limitation
Traditional quantitative reverse transcription PCR	cDNA synthesized via reverse transcription; PCR amplification using circRNA-specific divergent primers targeting BSJs	Quantitative; low cost; easy to operate; suitable for targeted circRNA detection	Low throughput; risk of primer dimer artifacts
Short-read short sequencing	Captures total RNA, removes rRNA, sequences cDNA, and identifies circRNAs via BSJ alignment using tools	High throughput; unbiased; quantifies global circRNA profiles	False positives; requires RNase R treatment to enrich circRNAs; complex bioinformatics analysis
Long-read long sequencing	Uses reverse transcriptase's strand replacement activity for roll-over reverse transcription of circRNAs; amplifies via rolling-circle amplification to generate full-length circRNA-derived cDNA	High accuracy; resolves circRNA structure	Costly; low throughput; requires specialized equipment
Spatial transcriptomics	Designs probes targeting circRNA-specific BSJs; uses fluorescence *in situ* hybridization to bind circRNAs in intact tissues and cells	Enables spatial localization of circRNAs; distinguishes circRNAs from linear RNAs; preserves tissue morphology	Low sensitivity; semi-quantitative; labor-intensive
Single-cell circRNA sequencing	Combines single-cell RNA sequencing with circRNA-specific analysis pipelines; identifies circRNAs from full-length single-cell transcriptome datasets	Resolves cellular heterogeneity of circRNAs; detects cell-type-specific circRNA expression	Costly; requires large single-cell cohorts; data analysis is computationally intensive
CRISPR-Cas13 system-based circRNA electrochemical method	gRNAs target circRNA BSJs; CRISPR/Cas13a binds and cleaves circRNAs; electrochemical signals quantify cleavage products	Ultra-high sensitivity; high specificity	Requires specialized electrochemical equipment; complex probe/gRNA design; low throughput
Droplet digital PCR	Partitions samples into ∼20,000 oil-in-water droplets; each droplet undergoes PCR amplification of circRNA BSJs; counts positive droplets for absolute quantification	Ultra-high sensitivity; absolute quantification; high precision	Low throughput; high cost per sample; limited to targeted circRNA detection
Electrochemical/optical nanobiosensors	circRNA-specific probes bind targets; signal amplification enhances detection signals	Rapid detection; suitable for liquid biopsies; high specificity	Sensors require optimization for stability; they may be affected by body fluid matrix interference
Echo crosslinked DNA nano-aggregates	Palindromic DNA sequences form nano-aggregates that specifically bind circRNA BSJs; isothermal amplification generates detectable signals	Rapid detection; low detection limit; distinguishes cancer stages	Requires complex DNA nano-aggregate synthesis; not validated in large clinical cohorts; high manufacturing cost

**Spatial transcriptomics:** The circFISH technology is based on the principle of fluorescence *in situ* hybridization, which designs probes across reverse splice sites that specifically bind to the circular structure of circRNAs without binding to linear RNAs, enabling the spatial localization of circRNAs within tissues. By tapping into the dynamics, tissue specificity, and spatiotemporal resolution of circRNAs, they give them a unique advantage over traditional biomarkers and break through the traditional static marker thinking.^87^

**Single-cell circRNA sequencing:** Recently, researchers have combined the specific circRNA analysis process with single-cell RNA-sequencing technology, based on massive single-cell full-length transcriptome sequencing datasets, to achieve efficient identification and deep mining of circRNAs at single-cell resolution, and to explore the cellular heterogeneity of circRNAs in depth, which revealed the potential of the application of circRNAs as cell type markers.^88^ In addition, the biomarker value of individual circRNAs is limited, and joint analyses integrating genomic, transcriptomic, proteomic, and other multi-omics data are expected to reveal the regulatory networks of circRNAs in diseases.

### Innovations in nucleic acid molecular detection technology

**CRISPR-Cas13 system-based circRNA electrochemical method:** By designing gRNAs targeting the reverse splice site of circRNAs and using the CRISPR/Cas13a system to bind to circRNA-specific back-spliced junction sequences, circRNAs can be specifically recognized and cleaved while avoiding the interference of homologous linear RNAs, thus enabling accurate quantitative analysis.^89^^,^^90^ Recently, Sha et al developed a circRNA electrochemical method based on the CRISPR-Cas system using an isothermal primer exchange reaction as a bridge, combining the unique circRNA targeting ability and the cascade inverse cleavage activity of Cas effectors. The detection limit of circRNA was as low as 2.13 × 10^−15^ m, enabling highly specific circRNA molecular diagnosis.^91^ However, standardization of gRNA design and assay conditions across laboratories remains a challenge for clinical implementation.

**Droplet digital PCR:** This PCR is a highly sensitive and accurate quantitative method that utilizes water-in-oil emulsion technology to partition samples into approximately 20,000 nL-sized droplets, each serving as an independent reactor for nucleic acid amplification. This partitioning enables precise absolute quantification, especially advantageous for low-abundance targets. An automated droplet detector then records the fluorescence signals of the droplets, allowing highly sensitive quantification of low-abundance circRNAs, especially for microdetection in clinical samples.^87^ Wang et al developed a detection technique combining the CRISPR-Cas13 system with droplet digital PCR that can realize quantitative detection of circRNA as low as 10 aM under amplification-free conditions, showing extremely high sensitivity.^92^

### Innovations in nanotechnology and biosensors

**Electrochemical/optical nanobiosensors**: Rapid detection is achieved by designing circRNA-specific probes combined with signal amplification techniques. As a liquid biopsy approach, the detection of circRNAs in blood has been utilized for lung cancer screening, offering a novel tool for early diagnosis and prognostic assessment. A novel optical nanobiosensor, leveraging surface-enhanced Raman spectroscopy and catalytic hairpin assembly, has been developed to precisely detect circSATB2 in blood. As this circRNA is critically involved in lung cancer progression, the method paves the way for improved early screening and treatment monitoring.^93^

**Echo crosslinked DNA nano-aggregates:** Isothermal detection of circRNA in 30 min with a detection limit of 77.56 fM, along with high specificity and rapid response, which is outstanding in the precision diagnosis and treatment of diseases, and provides a new solution for the immediate detection of circRNA. A system of gyroscopically cross-linked DNA nano-aggregates developed from this platform permits rapid circRNA detection and accurate staging/subtyping of lung cancer, holding great promise for early screening applications.^94^ However, the combination of nanomaterials and biosensor technology still needs to be further optimized to improve the stability and reproducibility of the sensors and to reduce the cost of the assays to suit the needs of clinical applications. However, these emerging technologies face limitations in accessibility and standardization. Nanomaterial-based sensors require further optimization to improve stability and reproducibility while reducing costs for clinical suitability.

## Artificial intelligence (AI)

AI-based algorithms have been used to predict circRNA expression, providing new tools in the spatiotemporal regulatory mechanisms of circRNAs during disease and development. The cellular heterogeneity of circRNAs is directly predicted by deep learning modelling the expression levels of cis-acting elements over trans-regulators.^85^^,^^95^ Meanwhile, AI algorithms show great potential for expression data complementation and structure prediction. Our group identified circulating cZNF292 as a potential biomarker for acute myocardial infarction and subsequently developed a “CM + cZNF292” prediction model using a machine learning workflow that incorporated LASSO regression with 10-fold cross-validation and logistic regression, with performance evaluated by ROC analysis. This work provides a reference for integrating artificial intelligence with circRNA research in cardiovascular disease applications.^96^ Nevertheless, circRNA is not directly applicable to existing models due to its unique circular structure and exon rearrangements, and we need to further develop proprietary training sets and modelling strategies. In addition, the co-diagnosis of circRNA with traditional biomarkers and imaging tests, combined with deep learning algorithms to build the model, may break through the traditional diagnostic model and be more accurate and efficient, enabling individualized precision medicine.^97^ Future research needs to combine machine learning to mine the interaction networks of coding RNAs and proteins, the construction of disease-specific molecular profiles, the development of standardized testing processes, and unified databases to address the discrepancies between different research results, and thus improve the predictive accuracy of circRNA as a biomarker for human diseases.^98^ However, a major limitation in this area is the lack of large-scale, well-annotated datasets. Current public databases often suffer from small sample sizes, incomplete clinical annotations, including missing critical patient information such as age and comorbidities, and inconsistent methodologies for circRNA detection. These limitations frequently lead to overfitting and poor generalization of AI models. Training reliable models requires integrating standardized circRNA expression data from multiple centers along with comprehensive phenotypic and genotypic annotations.

## Clinical translation process of circRNA biomarkers

Given the considerable promise of circRNAs as disease biomarkers, researchers are actively pushing their translation from bench to bedside. The core challenge of circRNA clinical translation is the low abundance expression of circRNAs in tissues and body fluids, and how to achieve accurate, efficient, and rapid detection, and reduce the cost of detection has been one of the difficulties in clinical translation. In addition, the enrichment efficiency of circRNA in different body fluids. EVs, as the messengers of organ or cell-to-cell communication, are stable and not easy to degrade by the EVs that carry circRNA; however, there is not yet a standardized and unified method of extracting EV-circRNA.

The complexity of circRNA biology and the contradictory roles of the same circRNA in different diseases, such as circHIPK3 promoting tumor in cancer and playing a protective role in myocardial injury,^99^^,^^100^ as well as the spatial and temporal dynamics of circRNA expression regulation in disease progression, such as the remodeling of circRNA profiles of drug-resistant clones after chemotherapy, require more basic and clinical research data to verify. Moreover, a single biomarker is prone to diagnostic errors, and the combination of multiple markers or marker(s) combined with imaging and genetic background information promotes personalized precision medicine. For example, the combination of the new circRNA biomarker circCCDC66 and the traditional biomarker CEA in the diagnosis of colorectal cancer has increased the AUC from 0.78 to 0.92, which greatly improves the accuracy of detection.^101^

In addition, an increasing number of circRNAs have demonstrated their translational potential as biomarkers through clinical studies. In a clinical study conducted at Shanghai Ruijin Hospital, circHAS2 could be used as a stratification marker for advanced CRC patients to guide amlotinib-targeted therapy and significantly reduce tumor load.^78^ Long-term arsenic exposure leads to increased risk of liver cancer. Researchers found that the level of circRNA_100284 in serum was positively correlated with the dose of arsenic exposure, which could be used as a marker for early cancer risk assessment, and epidemiological screening has been carried out in arsenic-contaminated areas for stratified management of high-risk populations.^102^ Based on this, the use of deep learning algorithms combined with aggregated association data between circRNAs and diseases database, Circ2Disease, and other circRNA clinical disease data to construct a machine learning model for circRNA disease association prediction may be a trend for mining disease biomarkers.

To successfully translate circRNA biomarkers into clinical practice, a coordinated strategy is essential, starting with prioritizing standardized pre-analytical protocols, including the use of EDTA plasma, sample processing within 2 h, and consistent column-based RNA extraction methods. Prospective clinical trials conducted across multiple independent centers are then critical to confirm the clinical utility and generalizability of circRNA biomarkers, as such large-scale validation helps establish robust performance across diverse populations and clinical settings; promising candidates should be validated through these large-scale, multi-center cohorts, analyzed alongside established biomarkers to strengthen diagnostic accuracy. Concurrently, future efforts must be directed toward developing low-cost detection tools and establishing standardized reference genes, ensuring both accessibility and reproducibility. Furthermore, regulatory approval for biomarkers demands extensive evidence of analytical validity, clinical validity, and clinical utility, as stipulated by agency guidelines. Ethical aspects, including informed consent for biomarker testing, data privacy protection, and equitable access, also need to be integrated into the development process. Finally, integrating AI with comprehensive clinical databases will enable the construction of disease-specific predictive models to support early screening and personalized risk assessment. Future, more clinical research data are needed to advance the clinical translation process of circRNA biomarkers (Fig. 4).

**Figure 4:**
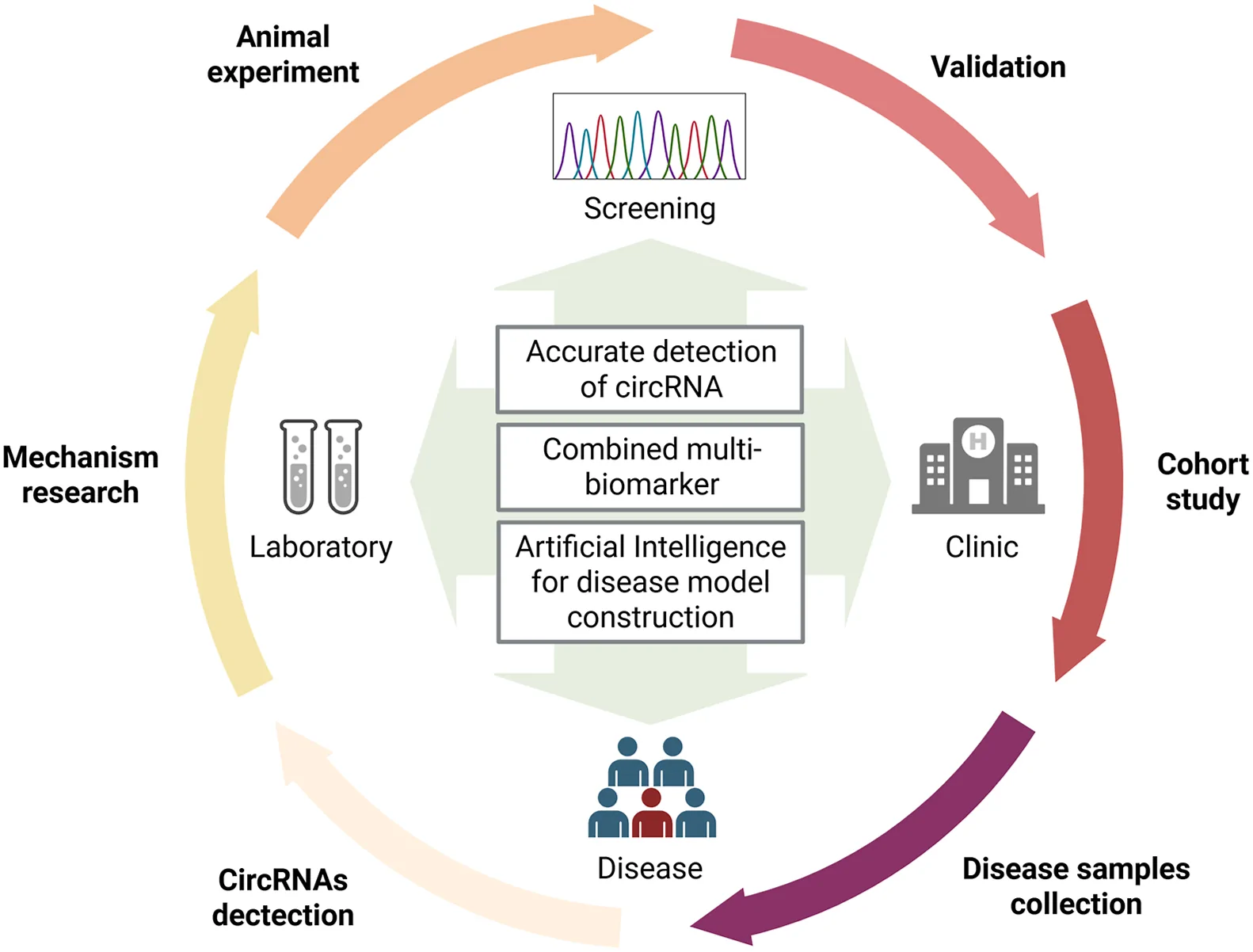
Workflow of clinical translation of circRNA biomarkers. This diagram illustrates the workflow from screening for differential circRNAs in disease samples, to experimental studies to validate the mechanism of circRNA action, and clinical cohort studies to validate the potential of circRNAs as biomarkers. The figure was created with BioRender.com, with permission.

## Conclusions and future perspectives

There is growing interest in circRNAs due to their diverse cellular functions, implications in various diseases, and high stability conferred by their covalently closed-loop structure, highlighting their clinical utility for diagnostic and prognostic development. Recent years have witnessed the emergence of innovative methods for circRNA identification, functional analysis, and therapeutic exploration. However, substantial translational research is still required before circRNAs can be established as robust clinical biomarkers for human diseases. In this review, we summarize and update the circRNA research between 2018 and 2025, focusing on the progress of circRNA research based on different human diseases, the innovations in circRNA detection technologies, and the current status and challenges in advancing the clinical translation of circRNA as a biomarker, in the hope that this information can provide researchers with a reference to the translational direction of circRNA research. It is hoped that this information will provide a reference for researchers in the direction of circRNA research and translation.

CircRNAs are stably present in blood, urine, cerebrospinal fluid, and other body fluids, making them ideal candidate molecules for non-invasive liquid biopsies. However, the low abundance of circRNAs and the difficulty of detecting them make them challenging to translate into the clinical setting. In recent years, advances in sequencing technologies such as spatial transcriptomics and single-cell sequencing have endowed circRNAs with novel biomarkers of dynamics, tissue specificity, and spatiotemporal resolution, breaking through traditional static marker thinking. Combined multi-omics testing, or combined traditional biomarkers, has significantly improved the accuracy of diagnostic testing. The rise of AI, combined with clinical database information, has enabled building disease prediction models capable of early disease screening and individualized precision medicine. Besides, the intersection of medical and industrial disciplines, based on biosensing, to promote the translation of engineered circRNA sensor research, is expected to achieve real-time *in vivo* monitoring, dynamic monitoring of disease progression, and therapeutic efficacy. While circRNAs show considerable clinical potential and detection methods continue to evolve, their translation requires overcoming key obstacles. The sensitivity and specificity of candidate circRNAs must be confirmed through large-scale cohort studies to mitigate the pitfalls of single-marker reliance. Additionally, the prohibitive cost of high-throughput sequencing and nanotechnology limits accessibility, driving the need for affordable and efficient diagnostic platforms.

CircRNAs play a pivotal role in disease pathogenesis, demonstrating considerable potential not only as diagnostic biomarkers but also as therapeutic targets for a variety of human diseases. It is now possible to synthesize circRNAs artificially *in vitro*, for example, by synthesizing circRNA aptamers with special double-stranded structures *in vitro* and delivering them via nanoparticle encapsulation, thus realizing RNA therapy.^103^^,^^104^ In addition, circRNAs are not dependent on cap translation, are highly stable in structure, and do not require nucleotide modification for activity, making circRNA vaccine development also of great potential.^105^ However, circRNAs hold promise for clinical translation, but require further rigorous validation and standardized detection platforms.

Overall, circRNAs are promising biomarkers for diagnosing and prognosticating human diseases, owing to their inherent stability, specificity, and evolutionary conservation. However, their successful translation into clinical practice requires further translational research. With the continuous development of bioinformatics tools, and in the future, relying on technological innovation and the multidisciplinary intersection of bioinformatics, nanotechnology, AI, and clinical medicine, circRNAs will gradually realize the leap from basic research to clinical application, and provide a new pathway for early diagnosis, dynamic monitoring, and precise treatment of human diseases.

## CRediT authorship contribution statement

**Yiqing Chen:** Writing – review & editing, Writing – original draft. **Shuting Guo:** Writing – review & editing, Writing – original draft. **Dongchao Lu:** Writing – review & editing, Writing – original draft. **Michail Spanos:** Writing – review & editing, Writing – original draft. **Xuan Ye:** Writing – review & editing, Writing – original draft. **Huihua Chen:** Writing – review & editing, Writing – original draft, Conceptualization. **Junjie Xiao:** Writing – review & editing, Writing – original draft, Funding acquisition, Conceptualization.

## Funding

This work was supported by the grants from the 10.13039/501100001809National Natural Science Foundation of China (No. 82020108002, 82225005 to J.X.), 10.13039/501100003399Science and Technology Commission of Shanghai Municipality, China (No. 23410750100, 21XD1421300 to J.X.), and the “Dawn” Program of Shanghai Education Commission (China) (No. 19SG34 to J.X.)

## Conflict of interests

Junjie Xiao is a member of *Genes & Diseases* Editorial Board. To minimize bias, he/she was excluded from all editorial decision-making related to the acceptance of this article for publication. The remaining authors declare no conflict of interests.
