# Supplementary material for: Circular RNAs as promising biomarkers for human diseases: An update for 2018–2025
Source: Genes Dis. 2026 Jan 19;14(3):102043. doi: 10.1016/j.gendis.2026.102043 (PMC13745043; doi:10.1016/j.gendis.2026.102043)
Supplement: Supplementary file 5 [file mmc1.docx]

##### Supplemental methods

###### Search strategy

The reviewers conducted a systematic and comprehensive literature search on circular RNAs and various human diseases. The databases searched included Pubmed and Embase, with keywords “circular RNA”, “circRNA”, etc. The search period time spanned from January 1st, 2018 to January 1st, 2025.

###### Inclusion and exclusion criteria

The inclusion criteria were: (1) Participants: Human patients or animal models with cancer, cardiovascular diseases, and other human disease, as well as disease-related cell lines. Studies must adhere to medical ethics and include appropriate control groups. (2) Intervention: Measurement of circRNA expression levels in tissues, plasma, serum, or other body fluids for biomarker evaluation. Any ethically conducted experimental or clinical intervention is permitted. Studies describing advances in circRNA detection methods are also included. (3) Outcomes: Studies reporting quantitative changes in circRNA expression, diagnostic accuracy metrics, mechanistic insights into circRNA function in disease or clinical translation applications. (4) Study design: Original basic or clinical studies that investigate the role of circRNAs as biomarkers in human diseases. Studies focusing on the development, comparison, or validation of circRNA detection methods are also eligible.

The exclusion criteria were: (1) Research that has been retracted; (2) Studies with poor study designs; (3) Studies with question research methods.

###### Literature screening and data management

The initial database search yielded a total of 6086 publications, which were imported into EndNote for management. After removing duplicates, the remaining records were screened by two independent reviewers. First, the titles and abstracts of all articles were assessed for relevance to the topic of circRNAs as disease biomarkers. Subsequently, the full texts of potentially eligible articles were evaluated based on the predefined inclusion and exclusion criteria. Any disagreements between reviewers were resolved through discussion until a consensus was reached. Articles with inadequate study design or insufficient data were excluded. Finally, a total of 1095 publications met all criteria and were included (Figure S1).

**Figure S1.** Flow diagram of literature screening. The flowchart illustrates the process of literature screening, from initial database searching through to the screening, selection, and final inclusion of eligible articles.
